# Book Reviews

**Published:** 1893-01

**Authors:** 


### Book Reviews.

SURGICAL DISEASES OF THE OVARIES AND FALLOPIAN TUBES, INCLUDING TUBAL PREGNANCY. By J. BLAND SUTTON, F. R. C. S., Assistant Surgeon to the Middlesex Hospital, London; late Hunterian Professor, Royal College of Surgeons, of England. In one 12mo volume of 514 pages, with 119 engravings and five colored plates. Cloth, $3.00. Philadelphia: Lea Brothers & Co. 1892.

Those who are acquainted with the literature of gynecology, and especially with the work Mr. Sutton has done, will not be surprised to learn that his latest book is one of the best on the subject with which it deals. There is, perhaps, as much mystery connected with the function of the ovary, and especially with its reproductive agency, as with any other organ in the human body. Its functions in health are attuned on a most delicate scale, and its peculiarities in disease are marvelously intricate and manifold.

Of all the diseases to which the ovary is liable, perhaps there is not one more curious or of greater interest to the majority of physicians than dermoid cysts. The contents of these cysts are so variable and their origin so obscure, that it furnishes an interesting field for the pathologist in which to exercise his knowledge and skill. Mr. Sutton has given a succinct account of these curious tumors, but we are unable therein to discover any new light on the subject. Solid tumors of the ovary are dealt with interestingly and instructively; so, too, are ovarian tumors in infancy and childhood.

The part on ovarian cysts and ovarian hydrocele furnishes an interesting study, but one of the most absorbing subjects is the coëxistence of ovarian cysts and pregnancy. Formerly it was thought unadvisable to meddle with cystic tumors of the ovary during pregnancy, and the law was to deal with the pregnancy itself when it advanced to a point of danger, rather than with the tumor. Under the improved methods of the present day, together with the knowledge of the fact that the ovary itself does not influence the progression of pregnancy, abdominal surgeons hesitate not to remove even both ovaries during the early months of pregnancy, with the absolute expectation that the mother will go to term, and be delivered of a living child. A sufficient number of double ovariotomies have been performed during pregnancy, where all these conditions have been obtained, to justify this as the only rational procedure.

Part III. of Mr. Sutton's excellent book is taken up with the subject of Tubal Pregnancy. Here, again, is a most interesting field for physiological research. The surgical questions relating to this disease are as nearly settled as they can be for the present, and mainly resolve themselves into the fact that, as soon as a diagnosis of tubal pregnancy can presumptively be made, an abdominal section is required for the safety of the patient It is no longer a question of delay or tentative measures ; electricity itself has ceased to be thought of in connection with these cases, and injections of lethal agents into the sac has failed to command any further attention in society debates or journalistic articles. At a recent meeting of one of the foremost National special societies, the subject of electricity in connection with ectopic pregnancy did not enter into the debate at all. So we feel justified in saying that upon a presumptive diagnosis of tubal pregnancy the patient should be advised to submit to an abdominal section at once. Delays, always dangerous in critical conditions, are doubly so here ; for a woman with this direful malady may not know at what moment her life will be swung over the abyssmal chasm of death, and held by so slight a thread that one single error of action may prove fatal. This subject has been so thoroughly discussed in the JOURNAL within the last two or three years, that we do not feel called upon to express anew our opinions on its pathological points, but we desire to reaffirm at this time those we have heretofore set forth.

The final section of Mr. Sutton's book deals with methods of performing operations for ovarian and tubal diseases. They are mainly those understood and carried out by the principal abdominal and pelvic surgeons, both in this country and in Europe. We are impressed with the straightforwardness of Mr. Sutton's style, and with its conciseness, as well as, in some instances, his original method of dealing with the various branches of his subject. His book will easily become adopted as a standard in the medical schools, and will serve to aid in the delineation to students of the intricate and absorbing subjects with which it deals. No specialist can consider his library at all complete unless this book is found on its shelves, and many other physicians interested in the subjects on which it treats will make haste to possess it.

A future edition, that, no doubt, will soon be demanded, will probably present the subjects on a much larger scale and with more elaborate illustrations.

---

TUBERCULOSIS OF BONES AND JOINTS. By N. SENN, M. D., Ph. D., Professor of Practice of Surgery in Rush Medical College; Professor of Surgery in the Chicago Polyclinic; Attending Surgeon Presbyterian Hospital; Surgeon-in-Chief St. Joseph's Hospital; President of the American Surgical Association; President of the Association of Military Surgeons of the National Guard of the United States; Permanent Member of the German Congress of Surgeons, etc. Illustrated with 107 engravings (seven of them colored). In one handsome royal octavo volume. 520 pages. Extra cloth, $4.00 net; sheep, $5.00 net; half-Russia, $5.00 net. Philadelphia: The F. A. Davis Co., Publishers, 1231 Filbert street.

This book treats of a science that has only lately developed into knowledge. The author, in his first chapter, truthfully asserts that no department in medicine or surgery has witnessed a more radical change than the etiology of tuberculosis of the bones and joints. When we recall the fact, as also stated by the author, that the first anatomical demonstration of the identity of the process of synovial membrane in some cases of tumor albus, with tubercular lesions of the lungs, was furnished by Rokitansky in 1844, and that it was not until 1869 that Koster furnished convincing proof that miliary tubercle can be found in every fungus joint, we can appreciate what a modern science Senn has set forth in this work.

Taken all in all, there is no more fascinating surgical question than that relating to diseases of the bones and joints. Bone surgery is a most tempting field for the surgeon, for one particular reason perhaps, namely, that he is able to demonstrate the science of diagnosis and his own operative skill with more precision and perfection than can be done in any other branch of surgical art. When a surgeon reaches forth his strong arm and rescues a little cripple, he does something to benefit humanity, and deserves its grateful thanks.

Probably few men, if any, are more competent to discourse upon this subject than Senn, and, surely, no one has yet undertaken to prepare a treatise that in any manner rivals the one before us. A correct diagnosis lays the foundation for successful treatment, and this aphorism is found nowhere to fit more snugly than in relation to bone and joint disease. Until the nature of the disease is understood, nothing can make progress in the way of successful management. The tubercular nature of chronic diseases of bones is not as universally admitted as it ought to be, and this book of Senn's will do much towards enlightening the professional world on the subject. While it is true that most surgical teachers and operators of experience understand this fact, namely, the tubercular origin of most chronic affections of bones and joints, yet they rarely see the cases first ; hence, it is necessary that the general practitioner, who resides in country, town, village, or hamlet, should become familiar with the subject.

This book is well printed in clear type, and has some illustrations that are excellent, but we think the author might have made his book more serviceable to the general practitioner if he had paid more attention to the pictorial side of the question. This is a subject capable of being well illustrated by handsome photographic reproductions of cases and specimens. But, of course, such illustrations require time and money. One of the difficulties of the present is, that the men most competent to write books have the least time to give to their elaborate illustration. We hope that Senn will publish, within a reasonably short time, a new edition of this book, amply and adequately illustrated, on the lines that we have indicated.

---

International Pocket Visiting List. Sixty patients each week. 1893. Arranged for the use of practitioners. By J. C. Wilson, M. D., Physician to the German Hospital, etc., etc. Philadelphia: J. B. Lippincott Company.

This new candidate for professional favor is arranged by a practical physician, and possesses all the advantages which experience and application can give to such a useful business pocket-book. It is handsomely bound in black, and its paper is of the finest quality. It is manifestly an easy contestant for the front rank, even among the old and favorite visiting lists that have been many years on the market. We commend it to the favor of such physicians as have not already supplied themselves with this indispensable help to simplify their daily practice records.

INTERNATIONAL CLINICS. A Quarterly of Clinical Lectures on Medicine, Surgery, Gynecology, Pediatrics, Neurology, Dermatology, Laryngology, Ophthalmology, and Otology. By professors and lecturers in the leading medical colleges of the United States, Great Britain, and Canada. Edited by JOHN M. KEATING, M. D., Philadelphia, Consulting Physician for Diseases of Women to St. Agnes' Hospital, Philadelphia; editor Cyclopedia of the Diseases of Children.—J. P. Crozer Griffith, M. D., Philadelphia, Clinical Professor of Diseases of Children in the University of Pennsylvania; Professor of Clinical Medicine in the Philadelphia Polyclinic.—J. Mitchell Bruce, M. D., F. R. C. P., London, England, Physician and Lecturer on Therapeutics at the Charing Cross Hospital.—David W. Finlay, M. D., F. R. C. P., Aberdeen, Scotland, Professor of the Practice of Medicine in the University of Aberdeen; Physician to and Lecturer on Clinical Medicine in the Aberdeen Royal Infirmary; Consulting Physician to the Royal Hospital for Diseases of the Chest, London. January, 1892. Philadelphia: J. B. Lippincott Company. 1892.

The preface to the fourth volume of this periodical consists of a memorial of Charles Theodore Parkes, M. D., of Chicago. Dr. Parkes was a distinguished surgeon and one of the strong characters in the country, but we are at a loss to know why a volume of clinical lectures, by authors residing in various countries, should be especially chosen for the publication of this memoir. Its fit place would seem to be in the Transactions of the American Surgical Association.

We find, in this volume, the usual subjects of interest that have pervaded its predecessors. They are pretty well divided between the several departments, and will command attention according to the various preferences and desires of readers. Dr. Charles G. Stockton, of Buffalo, has a short lecture entitled, Diseases of the Pancreas; Carcinoma of the Pancreas, Kidneys, and Liver. These are all important subjects, and Dr. Stockton is most competent to deal with them, but we think five pages is rather too narrow a space in which to treat of such large subjects. However, we presume future lectures will be added in the series, which will further serve to complete the subject.

Dr. Joseph M. Mathews, of Louisville, speaks interestingly of Fistula-in-ano, but we think he is putting it too strongly when he says that he knows "no dressing in surgery that can compare with iodoform for freshly made wounds." Our observation is that iodoform has a better place in granulating and sluggish wounded surfaces than in fresh incisions. If these clinics were illustrated more completely, it would add very much to their interest. They are handsomely printed on excellent book paper, and deserve to be more fully illustrated. The fact is, we presume, that most of these lectures are obtained under great difficulties. The haste in which they are prepared, and other embarrassments that stand in the way of the publishers in obtaining them, make it difficult to present with that degree of perfection which is desirable. We would suggest, however, that more pains be taken to obtain good and complete illustrations from those clinicians who will take interest in preparing and illustrating their lectures, and less endeavor be made to cover such a wide extent of territory or subjects.

---

TEXT-BOOK OF THE ERUPTIVE AND CONTINUED FEVERS. By JOHN WILLIAM MOORE, B. A., M. D., M. Ch., University of Dublin ; Fellow and Registrar of the Royal College of Physicians of Ireland ; Physician to the Meath Hospital, Dublin ; Joint Professor of Practice of Medicine in the Schools of Surgery of the Royal College of Surgeons of Ireland ; Consulting Physician to the Cork-street Fever Hospital, Dublin, and to the Whitworth Hospital, Drumcondra ; Ex-Scholar and Diplomat in State Medicine of Trinity College, Dublin. William Wood & Co., Medical Publishers, New York. 1892.

It is interesting to know that the study of fevers is taking strong hold upon the profession of medicine. Instead of being obliged to look through the voluminous pages of a treatise on practice, as was formerly the case, we now can readily refer to monographs on the subject that give it more satisfactory exposition. Murchison, Hilton, Fagge, and Alexander Collie, have all written admirably upon this subject, but Moore says, with great force and truthfulness, that notwithstanding all these men have written, full justice has not up to the present been done to this important and fascinating theme. He further states, and we quote this time *verbatim :* "While I cannot pretend to have adequately supplied an admitted want in medical literature—namely, a reliable and comprehensive text-book of the eruptive fevers, yet I have endeavored to focus in these pages the most recent views on the etiology, bacteriology, symptoms, pathology, and treatment of this group of maladies." This seems to be the sum and substance of the whole matter. In a work of such vast comprehensiveness, there need be no doubt in the minds of anyone that the subject is amply and exhaustively treated. An examination of even a few pages of the book would convince the most exacting critic that the work had been skilfully and thoroughly done.

The tendency to resort to antipyretics as routine practice in almost all conditions of exalted temperature is so strong, that some will read with surprise the following which we quote at length:

The antipyretic remedies—as antipyrin (phenezone)—have no special action on the causes of fever. They lower the temperature by increased radiation of heat from the body, and they diminish heat production. They do harm by interrupting the course of the fever, diminishing the means of defense of the human organism, for a diminution in the production of heat is equivalent to a diminution in the vitality of the human organism and of its power of resistance. In excessive fever (hyperpyrexia), however, the cardiac muscles and the nervous system suffer, and under these circumstances a reduction of temperature is desirable, if, by abstraction of heat, heat production is not diminished.

The study of enteric or typhoid fever is, however, of the most absorbing interest, and this is shown by the fact that the last 150 pages of this book are devoted entirely to that subject. No more critical or exhaustive treatise on the subject can be found in the literature of medicine, and we do not believe that any physician engaged in active general practice can afford to deny himself the privilege of reading Moore's intelligent, modern, and exhaustive exposition of the subject.

---

A TEXT-BOOK OF MORBID HISTOLOGY FOR STUDENTS AND PRACTITIONERS. By ROBERT BOYCE, M. B., M. R. C. S., Assistant Professor of Pathology in University College, London. With 130 colored illustrations. Octavo; pp. xxiv.—477. New York: D. Appleton & Co., 1, 3, and 5 Bond street. 1892.

The study of pathology is constantly becoming more and more important, and more and more interesting as well; its importance progressing as science advances, and its interest keeping pace with its importance. A number of excellent works have appeared within the last few years, that aim to set forth the essentials for the study of histology; but, if any one has been published that equals in rank the one before us, we have not seen it. Mr. Boyce has been a student of Victor Horsley, and, very properly, follows the teachings of his master to a considerable extent. The introduction to the work, by Mr. Horsley, is a delightful tribute of master to pupil, and ought to be, as it no doubt is, treasured by Mr. Boyce as a most satisfactory endorsement of his work.

The book begins with practical directions for the study of histology and for the preparation of histological specimens. These are not very different, in the main, from the generally understood methods. However, they will repay careful study. The outlines of Inflammation, Repair, Degeneration, and Neoplasm occupy the next nine chapters of the book, and this is one of the most important sections between its covers. We have been especially interested in Mr. Boyce's exposition of the Infective Processes (chapter seventh), which is a most intelligent setting forth of the subject within our present lines of knowledge. The study of the histology of tumors, and especially of cancers, is an absorbing one, and nowhere will be found more satisfaction in its pursuit than here. The outlines of the Diseases of the Special Systems begin with chapter eleventh and continue to the end of the book, leaving only bibliography and the indexes to follow.

This work will, no doubt, be adopted in the schools and laboratories as a principal text-book on the subject of morbid histology, hence deserves to be examined with a critical eye. We have attempted to do so, and the result is that we have only praise to bestow upon it. The illustrations are beautiful specimens of colored reproductions from micro-photographs, that have been carefully and minutely executed. They serve to explain the text with a greater definiteness than any pictures we have ever examined in a text-book. Many of them convey almost as accurate a knowledge of the subject as could be obtained by personal examination of specimens through the microscope.

It ought to be said, with reference to the printing, that it is unusually well done, on extra book paper, in large type, with double leads, so that it is easy reading for even tired eyes. Any student of histology who does not make haste to familiarize himself with Mr. Boyce's work, is not doing justice to himself or to the science which he is attempting to master.

---

A Physician's Complete Book of Records. Call List, Record of Visits, Cash Accounts, Ledger, Obstetrical Record, Death Record, and General Memoranda, all complete in one volume. Edited and compiled by Samuel E. Walker, Ph. G., M. D. Philadelphia: Keystone Publishing Co. 1892.

It has always been difficult for a physician to deal with the subject of book-keeping, unless he delegated it to an expert. This course will answer only for such physicians as are engaged in an extensive practice, with no time to spare for the details of accounts. Within the last few years a variety of devices has been put forth, whereby the process of keeping accounts with patrons has been simplified, or, at any rate, an attempt in that direction has been made. The last candidate for favor is the work of Dr. Walker, which certainly possesses very many unique and apparently advantageous points, for which he claims superiority. We quote from his prospectus the following :

1. Every entry is an "original entry," and cannot be anything else ; therefore, meets all legal requirements in case of suit. 2. The name and address of each patient under treatment is under your eye each day ; no one can be overlooked. 3. Current charges for each day, for even a hundred visits can be entered in a very few minutes ; no fumbling over back pages ; your current work is all in one place before you. 4. No ledger is required ; each patient's account is complete, for each month, in one line ; there is no "posting" to be done. 5. From the nature of the method, each account must appear daily, exactly as it stands, and all in one place. 6. Accounts that are deemed "slow" or "doubtful," are conveniently grouped, so that they are constantly in sight, and, therefore, not forgotten and lost. 7. The time required for entering charges and credits will not exceed ten seconds a day for each patient under treatment. 8. No "cipher code" required. 9. Use may begin at anytime of the year, with no loss of space. 10. Each book contains space for 2,250 monthly accounts, each complete in one full line. 11. Complete double index, with space for addresses. 12. Arranged to record credits on any day of the year, as well as charges. 13. Absolute simplicity ; no explanation, whatever, is needed. 14. The ease and rapidity with which entries are made, *ensures their being made.* This point will be appreciated by the busy practitioner.

From an examination of the book, we are favorably impressed with it, and unhesitatingly recommend it to physicians who have not already supplied themselves with a simplified method of book-keeping. The price of this book, $5.00, including shipping charges, brings it within the range of the most modest purse.

---

THE PHYSICIANS' VISITING LIST (Lindsay & Blakiston's) FOR 1893. Forty-second year of its publication. Philadelphia : P. Blakiston, Son & Co. (successors to Lindsay & Blakiston), 1012 Walnut street. Sold by all booksellers and druggists.

This old favorite puts in its annual appearance for the forty-second time, and is as welcome as ever. It possesses the advantage of being the most compact and one of the most useful visiting lists in the market. It takes up less space and, at the same time, answers the purpose quite as well as most of the others. Its strong morocco binding in black, and its good paper with gilt edges, give it a substantial and handsome dress. The price is still from $1.00 to $3.00, according to the size and edition wanted.

A Manual of Physics. Being an introduction to the study of Physical Science. Designed for the use of University Students, by William Peddis, D. Sc., F. R. S. E., Assistant Professor of Natural Philosophy, in the University of Edinburgh. New York: G. P. Putnam's Sons. London: Baillière, Tindale & Co. 1892.

To a book of this nature, the rapid glance which we are necessarily compelled to bestow upon it, can hardly be expected to do full critical justice. However, even a cursory view is sufficient to discover that we have before us a work of uncommon value to the advanced student, for whose use it was especially intended. Even to those who have lacked the advantages of a collegiate education, but who desire to know the latest advances in the knowledge of physic, its many phenomena, and the laws which science has been enabled to deduce, its possession and study must be of great value. In our practical age, and especially in our own country, a want of knowledge of the scientific principles which are the foundation for every useful invention, whether in dynamics, optics, electricity, or other forms of physics, must necessarily be almost fatal to ingenious enterprises. This book is a small octavo, 496 pages, beautifully printed, well bound, and carefully indexed.

---

The Students' Handbook of Surgical Operations. By Frederick Treves, F. R. C. S., Surgeon and Lecturer on Anatomy at the London Hospital. In one square 12mo volume of 508 pages, with ninety-four illustrations. Cloth, $2.50. Philadelphia: Lea Brothers & Co. 1892.

Anything that emanates from the pen of Mr. Frederick Treves always receives, as it generally deserves, patient examination and careful reading. The present work, the author says in his preface, is intended for the use of students who are preparing for the final examinations, or who need a handbook to assist them in carrying out operations upon the dead body. It is an abridgment from this author's Manual of Operative Surgery, lately noticed in the JOURNAL. For the purpose indicated, we have not seen so complete a book in any department of medicine or surgery. It is a student's handbook, without being a quiz work adapted only and solely to the brain-cramming process. It is also a valuable aid to the student in the pursuit of surgical anatomy in the dead-room. The mechanical execution of the book is unusually handsome.

---

INDEX-CATALOGUE OF THE LIBRARY OF THE SURGEON-GENERAL'S OFFICE, UNITED STATES ARMY. Authors and subjects. Vol. XIII. Sialagogues-Sutugin. Washington: Government Printing Office. 1892.

This useful and voluminous publication has now reached a point where the end probably can be seen in the not distant future. It has been going on steadily for the past twelve years, and, one by one, these volumes have come as regularly as the autumn season has returned. The present volume contains 9,751 author titles, representing 4,213 volumes, and 6,806 pamphlets. It also includes 13,498 subject titles of separate books and pamphlets, and 29,896 titles of articles in periodicals. The total number of titles listed in the thirteen volumes now issued is as follows: Author titles, 147,329; volumes, 71,068; pamphlets, 126,806; subject titles of books and pamphlets, 141,782; journal articles, 423,704; portraits, 4,335. This will indicate to those not already advised the stupendous nature of the work, and the width of its scope. When finished it will make the most comprehensive reference series on medical subjects in the world.

